# Selecting Remote Measurement Technologies to Optimize Assessment of Function in Early Alzheimer's Disease: A Case Study

**DOI:** 10.3389/fpsyt.2020.582207

**Published:** 2020-11-05

**Authors:** Andrew P. Owens, Chris Hinds, Nikolay V. Manyakov, Thanos G. Stavropoulos, Grace Lavelle, Dianne Gove, Ana Diaz-Ponce, Dag Aarsland

**Affiliations:** ^1^Department of Old Age Psychiatry, Institute of Psychiatry, Psychology and Neuroscience, King's College London, London, United Kingdom; ^2^Big Data Institute, University of Oxford, Oxford, United Kingdom; ^3^Clinical Insights & Experience, Janssen Research & Development, Beerse, Belgium; ^4^Centre for Research & Technology Hellas, Information Technologies Institute, Thessaloniki, Greece; ^5^Alzheimer Europe, Luxembourg, Luxembourg

**Keywords:** Alzheimer's disease, dementia—Alzheimer disease, function, mild cognitive impairment—MCI, remote measurement technologies, telemedicine, activities of daily living

## Abstract

Despite the importance of function in early Alzheimer's disease (AD), current measures are outdated and insensitive. Moreover, COVID-19 has heighted the need for remote assessment in older people, who are at higher risk of being infection and are particularly advised to use social distancing measures, yet the importance of diagnosis and treatment of dementia remains unchanged. The emergence of remote measurement technologies (RMTs) allows for more precise and objective measures of function. However, RMT selection is a critical challenge. Therefore, this case study outlines the processes through which we identified relevant functional domains, engaged with stakeholder groups to understand participants' perspectives and worked with technical experts to select relevant RMTs to examine function. After an extensive literature review to select functional domains relevant to AD biomarkers, quality of life, rate of disease progression and loss of independence, functional domains were ranked and grouped by the empirical evidence for each. For all functional domains, we amalgamated feedback from a patient advisory board. The results were prioritized into: highly relevant, relevant, neutral, and less relevant. This prioritized list of functional domains was then passed onto a group of experts in the use of RMTs in clinical and epidemiological studies to complete the selection process, which consisted of: (i) *identifying* relevant functional domains and RMTs; (ii) *synthesizing* proposals into final RMT selection, and (iii) *verifying* the quality of these decisions. Highly relevant functional domains were, “difficulties at work,” “spatial navigation and memory,” and “planning skills and memory required for task completion.” All functional domains were successfully allocated commercially available RMTs that make remote measurement of function feasible. This case study provides a set of prioritized functional domains sensitive to the early stages of AD and a set of RMTs capable of targeting them. RMTs have huge potential to transform the way we assess function in AD—monitoring for change and stability continuously within the home environment, rather than during infrequent clinic visits. Our decomposition of RMT and functional domain selection into *identify, synthesize*, and *verify* activities, provides a pragmatic structure with potential to be adapted for use in future RMT selection processes.

## Introduction

The study of Alzheimer's disease (AD) symptomatology typically focuses on the progressive deterioration of cognitive functions, neglecting real-world translation of functional impairment. The Food and Drug Administration (FDA) guidelines defines the emergence of mild but detectable functional impairment as signifying the transition from stage 2 to stage 3 of early AD, with a diagnosis of overt dementia (stage 4) being made as the functional impairment apparent during stage 3 worsens (1). Moreover, the recent emphasis on disease modification in clinical trials has concentrated focus on the early stages of AD (2). Functional improvement is also a typical primary endpoint in AD clinical trials (3). In addition to its clinical and research relevance, function is more resilient to demographic and cultural confounding factors than cognition (4). Individual and population differences, such as ethnic minority groups, scale translation, and societal and cultural relevance can impact the efficacy and relevance of typical “pen and paper” tests of function, so it is imperative that sensitive and relevant measures are available to examine the most environmentally appropriate means of assessing function.

A commonly used scale to measure function is the “Alzheimer's Disease Cooperative Study-Activity of Daily Living” (ADCS-ADL) (5), which can predict progression to dementia (6). Other functional scales include, “The Progressive Deterioration Scale,” “AD Functional Assessment Change Scale,” “Neuropsychological Test Battery” (NTB), and “Interview for Deterioration in Daily Living Activities in Dementia.” More recent scales include, “The Everyday Cognition Scale” (ECog), measures daily manifestations of cognitive impairments in memory, planning, organization, language, divided attention, and visuospatial skills and is sensitive to mild cognitive impairment (MCI) subtypes (7), and the “Amsterdam Instrumental Activities of Daily Living Questionnaire,” can differentiate those likely to convert to dementia from MCI and subjective cognitive decline (SCD) (8). However, such scales are reliant on self or informant reporting rather than objective assessment, neglecting more complex areas, such as social functioning, despite social functioning, loneliness, and social isolation's contribution to dementia risk and morbidity (9–11).

The relationship between cognition, including executive processes, and function is crucial in AD research, especially at the prodromal stage. ADLs involve varying degrees of cognitive and executive load, depending on whether the ADL is basic, instrumental or advanced. In its original conception, MCI was defined by a decline in cognition but not in daily function, however, the consensus criteria for amnestic MCI has since been revised to encompass a minimal impairment in advanced ADLs (12), although subsequent studies (13, 14) and metanalysis report instrumental ADLs are also impaired at the MCI stage (15, 16). Executive function (17, 18) and memory (19, 20), rather than demographic factors, have been found to accurately predict ADLs and ADLs also map onto neuroimaging features, such as subcortical white-matter hyperintensities in aging populations without dementia (21, 22). Functional status measured by ADLs also has prognostic purposes, as those with MCI and mild functional impairments at baseline are more likely to convert to overt dementia (13, 23).

## Remote Measurement Technologies

The COVID-19 pandemic has heighted the need for remote assessment in older people, as they are at higher risk and are advised to minimize risk of infection by using social distancing measures, yet the importance of diagnosis and treatment of dementia remains unchanged. Advances in digital health, including electronic health records, portal technologies, and wireless communications, are likely to have a central role in future dementia assessment and care. Remote Measurement Technologies (RMTs), refers to, “any mobile technology that enables monitoring of a person's health status through a remote interface, with the data then either transmitted to a health care provider for review or to be used as a means of education for the user themselves” (24). RMTs may include a variety of sensors that detect changes in health status, offering a unique opportunity to accurately and continuously track and measure changes. RMTs can objectively, actively and passively collect numerous data points during everyday routines that include a variety of basic, instrumental and advanced ADLs. These datapoints can index symptom severity and progression, stability and regression, impact on daily life and response to treatment. Deploying RMTs to remotely capture signals related to function also offers the possibility of engaging people who would not normally want to participate in research and empowers patients by giving them an active and informed role in their own healthcare.

RMTs are gaining popularity in dementia research in measuring cognition (25) but for widespread implementation, it is critical that we use RMTs to measure relevant and sensitive variables that accurately, reliably, and objectively measure cognition and function. Due to COVID-19, older adults are recommended to use social distancing but such measures have the side-effect of reducing physical and social activity, as well as increasing loneliness and social isolation, all of which are associated with more rapid cognitive and functional decline (26). RMTs can collect valuable data on health status during such restrictions and provide an opportunity to shift how care and assessment is undertaken in dementia by deeply enriching information on disability, particularly function, where large datasets can be collected passively over several days, whilst patients go about their everyday routines, rather than relying on subjective anecdotal reporting, with its inherent biases.

RMTs are technically complex and, for most studies, it will be necessary to select technologies off-the-shelf, rather than engineer bespoke solutions. But, selecting RMTs from the marketplace is still a challenge. There is a broad spectrum of options, from those targeted at personal fitness and behavior change, through to research-grade data logging devices. Each represents a different attempt to balance compromises across data quality, technical reliability, and participant acceptability. Moreover, many manufacturers revise the hardware and software of their offerings annually, meaning published validation data, where it exists, rapidly becomes out of sync with what the marketplace can supply. Checklists, such as the Clinical Trials Transformation Initiative technology selection tool (27), listing important factors, can be used to ensure devices are evaluated thoroughly. These might motivate deep technical investigations such as, the review of developer application program interface (API) documentation, or empirical evaluation of current real-world sensor performance.

## Patient and Public Involvement

Whether designing health services, digital health technologies, or clinical research studies, it is widely accepted that participants' be part of the process. This could be through pilot studies, group-based stakeholder workshops, or participant representation on project steering committees. For RMTs, which inherently have some manifestation within participants' everyday lives, the need to understand participants' perspectives is especially important. In the Innovative Medicines Initiative 2 (IMI)-funded, “Remote Assessment of Disease and Relapse—Central Nervous System” (RADAR-CNS, https://www.radar-cns.org/), it is hypothesized that Human-centered Design (HCD) methods might be usefully adapted to the challenge of *selecting* RMTs, and a novel three-stage iterative process based on HCD principles has been proposed (28).

“Remote Assessment of Disease and Relapse-Alzheimer's disease” (RADAR-AD, https://www.radar-ad.org/) is a European Horizon 2020-funded multi-stakeholder public-private consortium exploring the potential of RMTs to improve the assessment of function in early AD. RADAR-AD is closely working with people affected by AD (coordinated by the patient organization, Alzheimer Europe) and regulators, selecting and, if needed, modifying the most relevant available devices and apps to sensitively measure early and clinically meaningful functional decline in early AD. The mapping of prioritized functional domains to RMTs has the potential to radically improve our ability to understand the very earliest stages of AD progression and predict deleterious outcomes, such as loss of independence or conversion to dementia, compared to current clinical assessments. Therefore, the main objective of this paper is to outline the processes through which the RADAR-AD consortium has;

- Identified relevant functional domains
- Engaged with stakeholder groups to understand participants' perspectives
- Worked with technical experts to select and evaluate relevant devices.

We systemically describe the procedures and rationale for the three separate but interrelated workstreams and how their outputs are amalgamated to provide the methodological and design framework for RADAR-AD.

## Methods

### Functional Domains Clinical Literature Review

We carried out an extensive literature review to select functions relevant to AD biomarkers, quality of life (QoL), rate of disease progression and loss of independence. We based the literature review on “The US Cognition Working Group of the Critical Path Institute's Personal Report Outcome (PRO) Consortium's” report to the Food and Drug Administration (FDA), which focused on advanced ADLs and interpersonal functioning. PubMed (https://www.ncbi.nlm.nih.gov/pubmed/) literature until March 2019 was searched using the keywords, “Alzheimer's disease,” “early Alzheimer's disease,” “activity of daily living,” “activities of daily living,” “basic activities of daily living,” “instrumental activities of daily living,” “advanced activities of daily living,” “interpersonal functioning,” “social functioning,” “functional impairment,” “functional status,” “mild cognitive impairment,” “MCI,” “prodromal Alzheimer's disease,” “preclinical Alzheimer's disease,” “presymptomatic Alzheimer's disease,” and “quality of life.” Studies are tabulated to guide the prioritization of these preliminary functional domains (Table 1).

**Table 1:** The functional domain selection process resulted in the identification of the following functional domains, sorted by relevance.

**Functional domain**	**Tier**	**Predicts MCI->AD conversion**	**Impaired in early AD**	**Predictive of decline**	**Reported by PAB**
1. Difficulties at work	1	x	x	x	x
2. Spatial navigation and memory	1	x	x	x	x
3. Planning skills and memory required for task-completion	1	x	x	x	x
4. Managing finances	2	x		x	x
5. Self-care	2		x	x	x
6. Self-management, e.g., running errands and shopping	2	x		x	x
7. Acquiring new skills	2		x	x	x
8. Sleep quality and circadian rhythms	2		x	x	x
9. Use of technology/devices	2		x	x	x
10. Dysnomia, word finding difficulties	3		x	x	
11. Gait	3		x	x	
12. Difficulties driving	3		x	x	
13. Interpersonal interaction	3		x		x
14. Motivation, signs of apathy or withdrawal	4				x

*Tier 1, highly relevant; Tier 2, relevant; Tier 3, neutral; Tier 4, less relevant*.

The search was then specified to sequentially include each of the individual ADLs and interpersonal functions from The US Cognition Working Group of the Critical Path Institute's PRO consortium report. Other studies were identified by reviewing relevant bibliographies in original papers. Clinical studies were included if the study participants had a confirmed diagnosis of AD (e.g., cerebrospinal fluid biomarkers) and used standardized instruments of evaluation. Functional domains where ranked and grouped by the empirical evidence for each to;

- i) Predict MCI-to-AD dementia conversion
- ii) Relevance to early AD
- iii) Being predictive of decline in people with dementia.

### RADAR-AD Patient Advisory Board Consultation

The results of the literature review were then handed over for discussion with the RADAR-AD Patient Advisory Board (RADAR-AD PAB). We particularly requested feedback on the relevance of the functional domain to the experience of having AD or caring for someone with AD. The RADAR-AD PAB was established at the beginning of the project by Alzheimer Europe, in collaboration with the project partners. The composition of the RADAR-AD PAB, its approach and work in the context of RADAR-AD has been described elsewhere (https://www.radar-ad.org/patient-engagement/patient-advisory-board). Many of the members are from an existing working group of people with dementia and had been involved in Public Involvement (PI) activities in the past. The RADAR-AD PAB provides advice and influences relevant decisions in the conduct of the project. The topic of functioning was addressed in the first meeting of the RADAR-AD PAB, in Luxembourg in March 2019. The session lasted 2 h and was facilitated by two members of Alzheimer Europe with experience in PI. Before the meeting, members of the RADAR-PAB received accessible information about the project, functioning, dementia and the issues that RADAR-AD wanted to address.

The first part of the consultation addressed the understandings of people affected by AD of the term “functioning” and how these could differ from or complement the way this is typically portrayed. A semi-structured discussion approach was used. For the prioritization exercise, members were presented with the functional domains identified in the literature review and asked for their views. The task consisted of sorting the identified domains into three different piles (labeled as “very important,” “fairly important,” and “not important”), based on their experience and according to the perceived importance of each of the different domains in the early stages of dementia. In addition, as the functional domains linked to social activities have been less frequently considered in the existing literature, members were specifically asked to consider any missing elements. The results from this search criteria were prioritized into tiers:

- Tier 1—Highly relevant
- Tier 2—Relevant
- Tier 3—Neutral
- Tier 4—Less relevant.

Functional domains that met all three established criteria (predicts MCI-to-AD conversion, relevance to early AD, being predictive of decline in people with dementia) and were reported as relevant by the RADAR-AD PAB were grouped into tier 1, functional domains that met two of the criteria and were reported as relevant by the RADAR-AD PAB were grouped into tier 2, functional domains that met one of the criteria and were reported as relevant by the RADAR-AD PAB were grouped into tier 3 and functional domains that met 1 of the criteria were grouped into tier 4. Interpersonal domains are less studied than ADL's in AD, resulting in these domains meeting fewer criteria than the basic ADL, instrumental ADL and advanced ADL functional domains during the indexing of this list.

### Remote Measurement Technology Selection for Functional Domain Measurement

Our RMT selection work consisted of three kinds of activity, which we refer to as:

- Identify
- Synthesize
- *Verify*.

In the first, we sought to develop a broad understanding of the landscape for selection, taking the functional domains and PAB perspectives, and augmenting with a review of relevant technologies. In the second, we created candidate RMT selections, each with detailed reasonings, and brought the best aspects of each together into a single proposal. In the last, we went into depth to ensure that every aspect of our rationale for selection was well-founded. These were overlapping activities, rather than strict sequential phases.

#### Identify Activity

Work on selecting RMTs was conducted by a group of academic and industrial experts within the technology work package of RADAR-AD. It began, independently from the work on functional domains selection above, by reviewing the available apps, wearables, and fixed home sensor technologies, and generating ideas of potential uses for these as measures of daily function. A targeted, focused literature review was conducted to identify a wide range of internet of things (IoT) wearable sensors and devices in elderly care (29). Most of those solutions used either custom-made sensors that are not available to procure or commercially available wearables that are equally as effective. Toward identifying a range of commercial solutions, prior RMT reviews, such as IMI ROADMAP (30) and the choices of IMI RADAR-CNS played a role, but equally as influential were the experiences of the team members themselves in deploying such devices in previous clinical studies. Progressing from a long-list of devices to a short-list was straightforward and based on clear-cut criteria, such as measurement capability, battery life, water resistance (people with dementia may forget to remove devices when showering or bathing) and cost. Throughout this *identify* activity, collaborative working with the clinical experts involved in functional domain prioritization provided incremental updates and ensured that devices were not prematurely removed from the selection process.

#### Synthesize Activity

Moving from a shortlist of RMTs, each with good qualities, to a final selection of devices, was considerably harder. Our technical experts were each asked to propose a specific selection of RMTs, with a description of the clinical protocol for their proposed use, and a rationale for how these devices mapped to the functional domains. Given the challenging and heterogenous nature of our domains of interest, it was no surprise to find that these proposals had very significant differences. Presenting and explaining these detailed proposals to the rest of the group provided a way to:

- Verify that they were technically feasible and correct
- Flush out novel solutions to the measurement challenge
- Unpack differing assumptions about what was clinically significant
- Compare our estimates of how acceptable each proposal would be to participants.

The strongest aspects of the best proposals were then combined to create a proposed final selection, and this was then iteratively refined until it could be agreed by the expert group. It was this “bringing together” which led us to refer to this part of our selection process as *synthesis*.

#### *Verify* Activity

There was a continual need to *verify* the information we were using to make decisions. Early in the process, we sought to verify technical claims made about various devices through in-depth examination of application programming interface (API) documentation, and evaluation of device outputs at the laboratory bench. A core feature of wearables and apps are measuring the number of steps a user takes based on their internal accelerometer sensor and then translating this to clinically valuable metrics such as physical activity levels, calories burned, sleep duration, depth, and interruptions. Therefore, we performed lab trials and assessed the accuracy of multiple wearables and apps in measuring “steps” as a core metric. As work progressed, we frequently needed to verify our understandings of what was clinically significant. Interactions with the clinical teams were therefore pivotal to the eventual choice of devices. During the process, we often used our prior experience to estimate the participant acceptability of our proposals. Periodically, we were able to engage with participants to verify their views. This was done initially through group-based evaluation with the RADAR-AD PAB and then later in dedicated workshop-based piloting. Participants in the workshop were presented with various device alternatives and rated them in terms of comfort, functionality, battery life, and price. They also addressed intrusiveness and privacy issues.

## Results

### Functional Domains

The full list of functional domains from the literature review is detailed in Supplementary Material 1 and functional domain feedback from RADAR-AD's PAB is detailed in Supplementary Material 2.

Table 1 prioritizes these results in order of significance of predicting MCI-to-AD conversion, relevance to early AD, being predictive of decline in people with dementia and being ranked as important by the RADAR-AD PAB. Based on our criteria of each functional domain's relevance, highly relevant functional domains are; “difficulties at work,” “spatial navigation and memory,” and “planning skills and memory required for task completion.” Relevant functional domains are, “managing finances,” “self-care,” “self-management,” “acquiring new skills,” “sleep quality and circadian rhythms,” and “use of technology/devices.” Neutral functional domains are, “dysnomia,” “word finding difficulties,” “gait,” “difficulties driving,” and “interpersonal interaction.” Functional domains of less relevance are, “motivation and signs of apathy or withdrawal.”

### Remote Measurement Technology Selection

Table 2 allocates *verified* RMTs to the prioritized list of functional domains. While most of devices refer to specific brands and models that fulfill the particular requirements needed (functionality, data types, access to data etc.), the Smart Home sensor category requirements can be fulfilled by a broad range of products [e.g., the FIBARO (https://www.fibaro.com/en/), Plugwise (https://www.plugwise.com/nl_NL/), or other Z-Wave-compliant product families (https://www.z-wave.com/)]. While wearables and Smart Home devices can unobtrusively monitor participants at home, other devices require a certain protocol or exhibit technological peculiarities that mandate use in a lab setting only. This includes the Banking App, which simulates automated teller machine (ATM) use on a tablet—proved to be an effective marker (31) and the GAIT measurement protocol.

**Table 2:** The device selection process identified remote measurement technologies that could capture digital signals from functional domains comparable to established measures of function.

**Functional domain**	**Existing measures**	**Potential digital measures**	**Selected technologies**
1. Difficulties at work	Amsterdam IADL	Brief daily app-based self-report or carer reports Sociometric wearable badges	Digitized Amsterdam iADL
2. Spatial navigation and memory	CDR Amsterdam IADL MMSE ECog ADCS-ADL FAQ	Gamification of navigational tasks GPS movement trajectories or deviation from navigation tools	Altoida Medical Device (https://altoida.com/) GPS (passive) RADAR-base app (passive)
3. Planning skills and memory required for task-completion	ADCS-ADL MMSE Amsterdam IADL ECog UPSA CDR	Phone app measures performance on gamified/virtual reality tests	Mezurio Altoida Medical Device Digitized Amsterdam iADL
4. Managing finances	ADCS-ADL UPSA ECog Amsterdam IADL SFS	Speed of resolving calculation exercises Speed of fulfilling “procedure” (i.e., filling out transfer form, authorizing transaction, etc.) Active tasks simulating banking activities	Banking app (31) Digitized Amsterdam iADL
5. Self-care	Amsterdam IADL UPSA ADCS-ADL CDR SFS	Carer uses smartphone app to report patients' self-care Smart sockets monitor domestic device use, e.g., kettle In-home movement sensors Smart tags monitor movement of key domestic artifacts e.g., fridge door Wearable cameras	Digitized Amsterdam iADL Oxford Metrics Group Autographer (passive) Smart Home Sensors for Presence, Appliance Usage, Open Door/Window (passive)
6. Self-management, e.g., running errands and shopping	ECog UPSA ECog NPI CDR Amsterdam IADL ADCS-ADL SFS	Phone app collects details of meals GPS data, deviations and accuracy of daily routine Gamified/virtual reality performance assessments	Digitized Amsterdam iADL Mezurio Smart Home Sensors for Presence, Appliance Usage, Open Door/Window (passive)
7. Acquiring new skills	CDR	Learning new gamified/virtual reality tests on a smartphone	Mezurio
8. Sleep quality and circadian rhythms	NPI Sleep Quality Index, Epworth Sleepiness scale	Mobile phone sleep tracker Wearable accelerometer or fitness tracker Wearable EEG headbands Bed-mounted or under-mattress sensors	Mezurio Fitbit Charge 3 (passive) Axivity AX3 (passive) DREEM Headband (https://dreem.com/en, passive)
9. Use of technology/devices	Amsterdam IADL MMSE ECog SFS	Frequency/duration and sophistication of smartphone use	Digitized Amsterdam iADL RADAR-base app (passive) Mezurio Altoida Medical Device
10. Dysnomia, word finding difficulties	MMSE	Active or passive analysis of speech and voice Keyboard dynamics	Mezurio
11. Gait	ADCS-ADL ECog	Dedicated gait sensors Smartphone-based walking test Fitness trackers	GaitUp Physilog Sensor (https://gaitup.com/physilog-sensor/) Fitbit Charge 3 (passive) Axivity AX3 (passive)
12. Difficulties driving	CDR Amsterdam IADL ECog	Smartphone GPS and accelerometer monitoring Driving diagnostic OBD2 data logger	Digitized Amsterdam iADL CANedge driving data logger (passive)
13. Interpersonal interaction		Wearable cameras Localized logging of nearby smartphone presence	Oxford Metrics Group Autographer (passive) RADAR-base app (passive)
14. Motivation, signs of apathy or withdrawal	SFS WHOdas 2.0 CDR MMSE NPI	App monitoring communication from/with phone Time spent in different locations Level of physical activity Social media use	Mezurio Oxford Metrics Group Autographer (passive) RADAR-base app (passive)

*ADCS-ADL, Alzheimer's Disease Cooperative Study/Activities of Daily Living scale; CDR, Clinical Dementia Rating; ECog, Everyday Cognition; FAQ, Functional Activities Questionnaire; MMSE, Mini Mental State Examination; NPI, Neuropsychiatric Inventory; SFS, Social Functioning Scale; UPSA, University of California San Diego Performance-Based Skills Assessment; WHOdas 2.0, World Health Organization Disability Assessment Schedule*.

## Discussion

This case study outlines the processes through which we identified relevant functional domains, engaged with stakeholder groups to understand participants' perspectives and worked with technical experts to select and evaluate relevant RMTs to measure function in early AD. Through a literature review and Delphi-type exercise, we identified and prioritized functional domains specific and sensitive to the early stages of AD progression and most predictive of deleterious outcomes, such as loss of independence or conversion to dementia that can be prioritized and targeted for RMT measurement. The input provided by members of the RADAR-AD PAB confirmed the great relevance of function for people affected by AD. Much of their discussions focused on the challenges that cognitive decline may pose to people who are working, as well as other complex tasks, which are part of daily life, such as finishing tasks or activities and managing the household or personal finances. Social activities and social life can also be greatly impacted and are areas that are particularly meaningful to people with dementia and carers. In addition, as functional domains are increasingly affected, measurement may become challenging, as the frequency of the activity may appear stable, but the quality and nature of functioning may have significantly deteriorated.

There is undoubtedly huge value in both the prioritized domains of function sensitive to the early stages of AD and in the selection of devices to measure them remotely. However, our selection of RMTs must be considered in relation to the specific requirements of the RADAR-AD project but they can be applied to any clinical studies that wish to employ RMTs. For example, the duration of our clinical study was important in deciding whether a device would be tolerable to participants, we selected devices that would fit within our budgetary constraints and, because RMTs exist within a fast-moving marketplace, any selection made today would need to be reviewed again by future projects.

The ongoing need for RMT selection makes reflecting on the process we followed especially relevant. We described our selection process as consisting of three inter-related activities. The *identify* activity, in which suitable devices were longlisted and then shortlisted, was relatively straightforward and drew on the team's existing experiences and technical skills. The structure of our project divided RMT selection from the identification of functional domains, but in retrospect it is clear that at least conceptually, these also fit well with the *identify* activity. Initially, the group made differing assumptions about how a device might be used or how options would be set-up based on our prior experience and in some cases, this led to discussion at cross-purposes. The antidote to this complexity was to ground each candidate selection of RMTs within a detailed proposal which outlined, not just a selection of complementary devices, but the exact clinical protocol for how each would be used, and the precise details of how it would be configured.

Despite being founded in such a comprehensive set of understandings, the *synthesize* activity was challenging:

- A complete selection would involve multiple devices and since each device often had multiple sensors, there was often more than one way to achieve a similar aim
- Because devices were subject to similar engineering constraints (of battery life or sensor hardware), there was often no ideal solution to meet our clinical or participant experience requirements
- Some devices contained detailed configuration options, for example, to trade-off measurement frequency with battery life
- A single device could be deployed within a number of different clinical data collection protocols (worn for a long or short period, during daytime only, or at night too, etc.) each with a different impact on participant acceptability and the clinical value of the data.

The synthesize activity involved not just bringing complementary devices together into candidate selection proposals, but in further bringing the best of these proposals together into a final selection. It had many of the characteristics of a problem-solving task. Like many RMT selection projects, we had to make trade-offs between desirable criteria, like breadth of sensors, user experience, battery life, and data quality. For example, we wanted a wrist-worn device with raw high frequency accelerometer data, with over 24 h of battery life, a heartrate monitor, which would be acceptable to participants, and provide them with some feedback. While not immediately obvious, in the end the best solution was to have two wrist worn devices, a research-grade device logging raw accelerometer data, Axivity AX3 (https://axivity.com/product/ax3), and a fitness activity tracker, Fitbit Charge 3 (https://www.fitbit.com/us/products/trackers/charge3), logging heartrate measurements. This introduced the additional burden of wearing two devices, but after consultation with participants, this was considered a much better option than a single device that offers both kinds of functionality at the cost of bulkier casing and a much shorter battery life. Polling the RADAR-AD PAB on the issue validated this choice in the framework of our *verify* activities.

While the *identify* activity delivered a broad view, our *verify* activity was a deep and focused attempt to ensure every aspect of our selection rationale was rigorously challenged. This was by far the most interdisciplinary aspect of RMT selection. Our very first *verify* activities were technical in nature. For example, one expert group member proposed heart rate variability (HRV) as a measure which could be supplied from a specific wrist-worn device. When this was checked against technical documentation, it became clear that, while HRV is used by the device within several proprietary algorithms (and as such, was legitimately mentioned within marketing materials) the HRV measure was not made available to third parties through its standard APIs. Our choice of wrist-worn wearable pivoted around this issue for some time. Alternative device selections that would allow HRV were proposed but would introduce compromises in user experience. The matter was ultimately resolved when we sought to verify the scientific value of HRV to our project, and as a result of discussions with the clinical team, we resolved not to measure it. This was typical of an issue that required debate to flow swiftly back-and-forth across disciplinary divides.

Although initially technical in nature, as we began to propose concrete selections of candidate RMTs, our *verify* activities were soon also highly concerned with participants' perspectives. Initially, we relied heavily on our combined expertise in RMT. For example, we often talked about our expectations of participant burden or experience, based on our prior experiences. We reasoned that because the Axivity AX3 wearable device had been acceptable to participants of UK BioBank (32), that it would also be acceptable to the participants of RADAR-AD. This kind of experience-based assumption was incredibly useful and helped us to proceed rapidly. However, as we neared final selection it became increasingly critical that we rigorously verify every component of the rationale that we had established to support our selection choices: Just because a device had been acceptable to one participant group (with a shorter wear duration and a younger demographic), did that mean it would also be acceptable to ours? Acceptability was consequently verified through presentation to the RADAR-AD PAB and evaluation in a participant workshop.

RADAR-AD has benefited from the work of RADAR-CNS, including their HCD inspired framework, which adapted elements from *de novo* design methodologies to the challenge of RMT selection. It is surprising that we have not ended up with a selection process that more closely followed this framework. One simple explanation for this would be to recognize the substantial work that went into adapting our process to match the specific *a priori* structure of our project. Making the most effective use of the expertise available, from technologists, clinicians, and participants alike, was our overriding priority and strength. We clearly were informed by principles from HCD (28) and of course by aspects of “design thinking” more broadly (33). But pragmatic adaptation of these techniques to fit our circumstances, maximize our strengths, address our weaknesses, and solve the problems we encountered, became more influential on the shape of our eventual process than an abstract model.

Inherent tension exists between techniques intended for *de novo* design and a process of selection. RMT selection is a critical part of the design of a clinical study where devices are used, and it is clearly appropriate to draw on design methodology. However, equally as clear, is that selection involves deciding whether a study should adopt the design decisions taken by others during the production of a candidate RMT; do *their* design decisions work in *our* context? Unlike *de novo* design, there is usually at least some evidence that a candidate off-the-shelf RMT did work in a related context. Within our selection process, it was frequently the case that such evidence was highly informative, and it would have been inefficient not to have used it, but as the final selection neared, we nevertheless needed to empirically verify whether those conclusions would truly hold within our own study. The *verify* activity is thusly named to acknowledge this perceived difference between *de novo* design and RMT selection, while emphasizing the need for rigorous evaluation.

## Conclusion

This case study provides a set of prioritized functional domains that are sensitive to the early stages of AD progression and a set of RMTs capable of targeting them. RMTs have huge potential to transform the way we assess function in AD, monitoring for change and stability continuously within the actual home environment, rather than during infrequent clinic visits. Technologies change rapidly and the ability to select the best RMTs is therefore critical. It is obvious that successful RMT selection must give equal weight to technical, clinical, and participant perspectives and this case study illustrates what such interdisciplinary working looks like in practice. Optimal selection is challenging. It must be broad to ensure no option is missed; it must also be deep to ensure every detail is correct and finding solutions may require solving problems that span disciplines. Finally, we decomposed RMT selection into three activities: *identify, synthesize*, and *verify*, which have potential to be adapted for use inside the selection processes of other projects.

## Author Contributions

AO and CH conceived the structure and content of the paper. AO, CH, NM, TS, GL, DG, AD-P, and DA provided revisions of the paper. DA was the coordinator of RADAR-AD. All authors contributed to the article and approved the submitted version.

## Conflict of Interest

DA has received research support and/or honoraria from Astra-Zeneca, H. Lundbeck, Novartis Pharmaceuticals, Biogen, and GE Health, and served as paid consultant for H. Lundbeck, Eisai, Heptares, Mentis Cura. The remaining authors declare that the research was conducted in the absence of any commercial or financial relationships that could be construed as a potential conflict of interest.
