# Supplementary material for: Selecting Remote Measurement Technologies to Optimize Assessment of Function in Early Alzheimer's Disease: A Case Study
Source: Front Psychiatry. 2020 Nov 5;11:582207. doi: 10.3389/fpsyt.2020.582207 (PMC7674649; doi:10.3389/fpsyt.2020.582207)
Supplement: Supplementary file 1 [file Table_1.docx]

Supplementary Material 1

**Supplementary table 1.** Results of literature review. 4MT = Four Mountains Test, AD = Alzheimer’s disease, ADL = activities of daily living, aMCI = amnestic mild cognitive impairment, CR = cognitive reserve, CVLT = California Verbal Learning Test, FAQ = Functional Activities Questionnaire, FTD = frontal temporal dementia, HC = healthy controls, MCI = mild cognitive impairment, pAD = probable Alzheimer’s disease, PPA = primary progressive aphasia, QoL = quality of life, SCD = subjective cognitive decline, TUG = timed up and go test

| Functional domain | Studies |  |
| --- | --- | --- |
| *Difficulties at work* | Myung et al, 2017^1^ | In MCI participants, the protective effect of high occupational attainment against cognitive decline disappears at the MCI stage, as AD conversion was higher in high attainers after controlling for potential confounders |
|  | Forstmeier et al, 2013^2^ | In older HCs, motivation-related occupational abilities were associated with reduced risk of MCI & reduced risk of AD in ApoE ε4 carriers but not in noncarriers |
|  | Boots et al, 2015^3^ | In participants at risk of AD, occupational complexity related to decreased hippocampal volume & increased whole-brain atrophy when paired with cognitive function, even after controlling for AD risk, vascular, mental health & socioeconomic factors |
|  | Garibotto et al, 2008^4^ | In pAD participants, MCI & HCs, there was a significant correlation between temporoparietal cortex, precuneus brain glucose metabolism & higher education/occupation in pAD & aMCI converters but not HC or non-converting MCI |
| *Spatial navigation & memory* | Tu et al, 2015^5^, Yew et al, 2013^6^ | Spatial navigation & orientation deficits have higher specificity than episodic memory tests in distinguishing AD from HCs & other dementias |
|  | Serino et al, 2015^7^ & Lithfous et al, 2013^8^ | Spatial disorientation is seen in early AD vs HCs |
|  | Mokrisova et al, 2016^9^ | In spatial navigation tasks in prodromal older cohorts, path integration is impaired |
|  | Laczó et al, 2011^9^ | aMCI ApoE4 homozygotes have poorer egocentric & allocentric spatial navigation than heterozygotes |
|  | James et al, 2011^10^ | Reduced daily life space relates to cognitive decline, risk of MCI & AD severity |
|  | Tung et al, 2014^11^ | Life space correlated with apathy & depression in AD vs HCs |
|  | Chan et al, 2016^12^ | 4MT scores correlated with hippocampal volume & precuneus cortical thickness. 4MT scores were significantly different between MCI biomarker+ & MCI biomarker- groups. 4MT test scores of ≤8/15 were associated with 100% sensitivity & 78% specificity for MCI biomarker+ |
|  | Moodley et al, 2015^13^ | 4MT was impaired in MCI & AD. 4MT score differentiated MCI biomarker + & MCI biomarker – subgroups: scores of ≤8/15 had 100% sensitivity & 90% specificity for detection of early AD |
|  | Kalova et al, 2005^14^ | Allocentric navigation is impaired in AD |
|  | Hort et al, 2014^15^ | Comparative to vs aMCI, AD participants failed to use start position or cues to navigate, whereas aMCI only showed allocentric deficits |
| *Planning skills & memory required for task-completion* | Baumard et al, 2018^16^ | AD participants have planning deficits comparative to HCs, semantic dementia & corticobasal syndrome. Performance on planning task predicted tool use in AD – whose deficit was generalised rather than mechanical |
|  | Schmitter-Edgecombe & Parsey, 2014^17^ | MCI participants produced more omission errors, inefficient action errors, substitution errors & irrelevant action errors vs older HCs & younger adult HCs on 8 ADLs, including outfit selection. Older HCs produced more inefficient errors than younger HCs |
|  | Lau et al, 2015^18^ Used the ECog & IADL Lawton & Brody, 1969^19^ | Planning & everyday memory predicted greatest risk of incident dementia in HC & MCI to define cognitive/functional markers of loss of independence in instrumental ADLs in MCI vs HC, Everyday Planning was associated with 3-fold increase in functional disability. |
|  | Farias et al, 2017^20^ | Using informant ratings in older HCs, the highest risk of MCI conversion was associated with increased baseline planning limitations. |
| *Managing finances* | Pedrosa et al, 2010^21^ | Balancing the cheque book was impaired in MCI |
|  | Barberger‐Gateau et al, 1999^22^ | In community-dwelling subjects, the ability to handle finances predicted dementia incidence at 3 years |
|  | Schmitter-Edgecombe & Parsey, 2014^17^ | MCI participants produced more omission errors, inefficient action errors, substitution errors & irrelevant action errors comparative to older adult HCs & younger adult HCs on 8 ADLs: including cheque writing |
| *Self-care* | Ashraf & Taati, 2016^23^ | Using wearable cameras to monitor handwashing trajectory discriminates between HC, MCI & AD |
|  | Buso et al, 2015^24^ | Wearing of GoPro [https://gopro.com/en/us/] identified 8 ADLs in a person with AD |
|  | Cortes et al, 2008^25^ | Over a 2-year period, AD participants showed greatest decline in dressing & bathing (via the Katz ADL Scale^26^). |
|  | Gillette-Guyonnet, et al, 2011^27^ | Over a 4-year period, AD participants showed greatest decline in dressing, continence & bathing |
|  | Schmitter-Edgecombe & Parsey, 2014^17^ | MCI produced more omission errors, inefficient action errors, substitution errors & irrelevant action errors comparative to older adult HCs & younger adult HCs on 8 ADLs, including outfit selection |
|  | Smit et al, 2016^28^ | In dementia residents, regardless of cognitive status, activity involvement in domestic tasks significantly related to care relationship scores, positive affect, restless/tense behaviour, social relations, & 'having something to do' |
| *Self-management* | Pedrosa et al, 2010^21^ | ‘Grocery shopping’ was impaired in MCI |
|  | McDermott et al, 2017^29^ | In older adults, memory resilience was predicted for males & females by younger age, higher education, stronger grip & everyday novel cognitive activity |
|  | Pedrosa et al, 2010^21^ | ‘Keeping appointments’ was impaired in MCI |
|  | Barberger‐Gateau et al, 1999^22^ | The responsibility for own medication, predicted dementia incidence at 3 years in 1582 community-dwelling participants |
|  | Schmitter-Edgecombe & Parsey, 2014^17^ | MCI participants produced more omission errors, inefficient action errors, substitution errors & irrelevant action errors comparative to older adult HCs & younger adult HCs on 8 ADLs: including ‘sweeping, ‘dusting’ & ‘outfit selection’ |
|  | Smit et al, 2016^28^ | In care home residents with dementia, regardless of cognitive status, activity involvement (including domestic tasks, cooking) significantly related to care relationship scores, positive affect, restless/tense behaviour, social relations, & 'having something to do'. |
| *Acquiring new skills* | Pedrosa et al, 2010^21^ | Initiating complex activities was impaired in MCI |
|  | Hedman et al, 2016^30^ | Using grounded theory in MCI participants to examine technology use, MCI related to 'downsizing doing', i.e., using technology in a new way, replacing technology with another, stop using technology, considering support from new technology in the present & future |
|  | Law et al, 2013^31^ | A newly designed functional task exercise programme aided cognitive functions & functional status in MCI |
|  | Lancioni et al, 2014^32^ | Using technology-aided programs in moderate AD, patients were presented with digital activity-relevant pictorial instructions. All patients exceeded 85% accuracy & were able to learn to activate music options |
|  | Vemuri et al, 2016^33^ | In both older HCs & MCI, in those with higher levels of education, increased midlife cognitive activity was associated with lower amyloid deposition in APOE4 homozygotes |
| *Sleep quality & circadian rhythms* | Beaulieu-Bonneau & Hudon, 2009^34^ | Up to 59% of AD participants had sleep disturbances |
|  | Hatfield et al, 2004^35^; Gehrman et al, 2005^36^ | Sleep-wake cycle disruption caused nocturnal waking & daytime sleepiness in moderate AD |
|  | van Someren et al, 1996^37^ | Better sleep-wake cycle was predicted by increased daytime activity & lower levels of daytime activity predicted poorer sleep-wake cycles in AD vs HC |
|  | Carvalho-Bos et al, 2007^38^ | Functional status & wellbeing related to lower nocturnal activity & higher daytime activity in female dementia participants |
|  | Otsuka et al, 1990^39^; Chen et al, 2013^40^ | Daytime blood pressure was higher in AD vs. HC. AD participants did not produce the nocturnal fall in blood pressure |
|  | Harper et al, 2004^41^ | AD circadian disruptions in core temperature increasingly deviated from HCs with progressive AD pathology (post-mortem Braak stage) |
|  | Volicer et al, 2001^42^ | Circadian disruptions in core temperature & motor activity were present in AD participants. Thermodysregulation related to sundowning severity |
|  | Most et al, 2012^43^ | AD participants had higher daytime proximal skin temperature vs. HCs. This elevation positively correlated with daytime sleepiness |
|  | Yamadera et al, 2000^44^ | Morning light therapy reinforced circadian rhythms in AD |
|  | Satlin et al, 1992^45^ | Evening light therapy improves sleep in AD |
|  | Hatfield et al, 2004^35^ | Sleep disorders & abnormalities occur before cognitive symptoms in AD |
|  | Prinz et al, 1982^46^ | AD patients have reduced REM sleep & sleep-wake cycle |
| *Use of technology/devices* | Jutten et al, 2017^47^ | Using the Amsterdam iADL Scale (A-IADL) in a pre-AD cohort (Aβ+ individuals with subjective cognitive decline) show differences across A-IADL, particularly use of technology |
|  | Stringer et al, 2018^48^ | Computer-based behaviours, e.g., mouse clicks, typing speed, pauses, corelated with cognitive scores in MCI vs HC |
|  | Hedman et al, 2018^49^ | Daily technology use became more challenging in years post-MCI diagnosis |
|  | Malinowsky et al., 2017^50^ | Daily technology use & subjective ability was significantly poorer in individuals with SCD vs HC |
|  | Couth et al., 2017^51^ | 21 key technology behaviours were identified as being sensitive to early cognitive impairment, e.g., text-based language use, incorrect passwords, mouse movements, difficulty opening correct item |
|  | Seelye et al, 2015^52^ | In MCI vs HC, frequency, pauses & efficiency of mouse movements discriminated between groups & correlated with cognitive scores |
|  | Austin et al, 2017^53^ | Unique internet search history items were identified as being associated with cognitive scores |
|  | Pedrosa et al, 2010^21^ | Telephone-use was impaired in MCI |
|  | Geda et al, 2011^54^ | Computer activity was reduced in MCI vs HC |
|  | Barberger‐Gateau et al, 2015^22^ | In 1582 community-dwelling participants, the ability to use the telephone predicted dementia incidence at 3 years |
|  | Anstey et al, 2013^55^ | Instrumental ADLs are most-effected by cognitive impairment over 8 years in older participants, included ‘difficulties making phone calls’ |
| *Dysnomia, word finding difficulties* | Ellendt et al, 2017^56^ | In a community-based sample, conversion to MCI was associated with language functions |
|  | Mesulam et al, 2008^57^ | In PPA participants, 7/11 logopenics had AD. Those with AD/PPA had entorhinal neurofibillary tangles, indicative of amnestic AD. AD pathology was not predicted by APOE4 genotyping |
|  | Nutter-Upham et al, 2008^58^ | Phonemic & semantic fluency were statistically (but not clinically) reduced in aMCI. Verbal fluency switching & production discriminated MCI from HC |
|  | Bracco et al, 1994^59^ | Over 9 years in pAD, dementia progressed more rapidly & severely in severely aphasic patients. Severe language disability was the strongest predictor of death |
|  | Bilgel et al, 2014^60^ | The first CVLT measure to decline was immediate recall, then delayed recall. As disease progressed, delayed recall & visual memory changed more rapidly than immediate recall. |
|  | Yotter et al, 2013^61^ | Pattern of amyloid deposition is related to cognitive data (via CVLT) & may be a biomarker reflecting total amyloid burden |
|  | Cosentino et al, 2006^35^ | In a 15-year longitudinal study of AD, higher verbal fluency scores at diagnosis predicted lower mortality |
|  | Tang-Wai & Graham, 2008^62^ | Language impairment in early AD involved spontaneous speech, paraphasic errors (semantic) & naming, & is impaired for less frequently used words/objects |
|  | Smit et al, 2016^28^ | In community-dwelling residents with dementia, activity involvement, including conversation groups, significantly related to care relationship scores, positive affect, restless/tense behaviour & social relations |
|  | Maneti et al, 2004^63^ | In young adult HCs, older adult HCs & AD participants, the only between-group difference in AD was an interference effect of combined semantic/grammatical information incongruence |
| *Gait* | Tung et al, 2014^11^ | In AD vs HC, life space correlated with steps & gait velocity |
|  | Nadkarni et al, 2009^64^ | In 4 groups: i) AD+subcortical hyperintensities (AD+SH), ii) AD-SH, iii) HC+SH, iv) HC-SH, HC-SH had a higher gait velocity. SH severity in frontal region & basal ganglia in AD & HC groups correlated with stride length & velocity |
|  | Ansai et al, 2017^65^ | In MCI, falls were associated with lower FAQ score, longer dual task test (TUG+motor-cog task) & higher depression. Falls in AD were associated with shorter turn2sit, walk test & visuospatial score. Shorter turn-to-sit phase was an independent predictor of falls |
|  | Pedrosa et al, 2010^21^ | *'Getting around outside the home'* was impaired in MCI |
|  | Mazoteras Muňoz et al, 2010^66^ | Using the TUG, 31.5% of AD participants had abnormal TUGs. 25.2% had impaired balance & 18.9% were gait impaired. Global TUT & gait impairments were associated with ADLs |
|  | Montero-Odasso et al, 2009^67^ | MCI participants’ gait was assessed. Under dual task (walking/counting backwards) conditions, gait velocity decreased & there was greater gait (stride time, step time) variability |
|  | Maquet et al, 2009^68^ | Under dual-task conditions (walking/counting backwards), MCI & AD displayed altered gait vs HC |
|  | Beauchet et al, 2011^69^ | Using the GAITRite system, aMCI had greater gait variability vs HC & non-aMCI |
|  | Montero-Odasso et al, 2012^70^ | Under dual-task conditions (walking/naming animals), MCI participants had increased gait variability vs HC from the same community |
|  | Muir et al, 2012^71^ | Comparative to HC, MCI & AD participants had decreased gait velocity, increased stride time & increased stride time variability during dual tasks (naming animals, serial subtraction by 7) |
|  | Sheridan et al, 2003^72^ | During free walking, gait variability was increased & walking was slower in probable AD vs HC. On dual task walking, gait speed decreased & variability increased. Gait variability was more effected than speed. Executive & cognitive scores were associated with increased gait variability on dual tasks only |
| *Difficulties driving* | Anderson et al, 2012^73^ | Neuropsychological tests significantly correlated with driving safety errors. Speed of processing, visuospatial processing, memory domains correlated with driving outcome measures |
|  | Fernandez-Romero & Cox, 2016^74^ | Using a specified virtual reality (VR) driving test (incorporating cognitive, visual & motor tasks), 'most' early AD participants were not fit to drive, unlike older HC. Comparative to HC, early AD had lower driving scores & lower event-related potentials to simulated self-movement peaking ~200ms after motion onset (n200) |
|  | Wadley et al, 2009^75^ | MCI participants had significantly poorer driving performance comparative to HC for global & discrete driving manoeuvres. Performance was also sub-optimal |
|  | Pedrosa et al, 2010 | Driving a car was impaired in MCI (n=30) |
|  | Paire-Ficout et al, 2018^76^ | Using in-car video recording, early-stage AD participants driving performance, including tactical self-regulation & critical events was impaired |
|  | Roe et al, 2018^77^ | Using the 12-mile modified Washington University Road Test, preclinical AD participants at stage 2 (+amyloid & tau scans) received more test fails or marginal passes than HC at combined 0 or 1 of staging. There were no cross-sectional differences in neuropsychological performance in passers vs failers |
|  | Ott et al, 2008^78^ | Over 3 years, early AD participants had more car accidents, driving offences & performed more poorly on a road test than age-matched HC. Although most passed the test at baseline, AD performance on the driving test deteriorated more severely over the 3 years than HC. AD progression, higher age & lower education related to increased likelihood of driving test failure/marginal pass |
|  | Hird et al, 2017^79^ | Using a driving simulator, driving errors was doubled in MCI, single domain aMCI, multi-domain aMCI vs aged-matched HC. multi-domain aMCI performed more poorly than HC & single domain aMCI. |
|  | Ott et al, 2000^80^ | In SPECT-confirmed AD or other dementia type, regional reduction of right hemisphere cortical perfusion (especially temporo-occipital area) related to poorer driving, as measured by caregiver ratings. Driving impairment & reduced frontal-cortical perfusion positively correlated. Clock drawing scores were associated with driving impairment |
|  | Ott et al, 2016^81^ | In aged-matched HC, AD & MCI, driving risk related to amyloid burden in all groups, even in the preclincal stage |
|  | Piersma et al, 2016^82^ | Neuropsychological assessment & driving simulator performance predicted on-road driving performance in patients with AD with 92.7% accuracy |
| *Interpersonal interaction* | Bora & yener, 2017^83^ | MCI related to impairments in facial emotion recognition (especially fear & sadness) & theory of mind |
|  | Lavenu & Pasquier, 2005^84^ | Compared to age, cognitive & disease duration matched clinical FTD control, AD patients performed better at test 1 than FTD. However, despite treatment, at test 2 (~40 months apart) AD participants showed a general worsening of emotion perception, whereas scores improved in FTD participants. Change in AD emotion recognition scores was not related to global cognitive scores |
|  | Phillips et al, 2010^85^ | Comparative to aged-matched HC & late-life mood disorder patients, emotion decoding in AD was more impaired, especially with subtle expressions. Emotion perception problems also predicted QoL |
|  | Albert et al, 1991^86^ | Comparative to HC, AD participants had poorer emotion perception. However, when cognitive scores were controlled for, the disparity was much smaller, indicating the role of cognition in emotion perception |
|  | Bediou et al, 2009^87^ | - Mild AD participants had poorer facial expression detection comparative to HC. aMCI did not differ from HC |
|  | Bertoux et al, 2015^88^ | AD participants’ emotion recognition of happy & sad facial expressions was impaired comparative to HC. Mild-AD/moderate-AD participants were significantly impaired vs HCs & very mild-AD |
|  | Zhang & Zhang, 1999^89^ | Not engaging in community activities was associated with risk of AD over 10 years |
|  | He et al, 2000^90^ | Elevated risk of dementia was associated with reduced community participation in older participants |
|  | Saczynski et al, 2006 | Social engagement in late life but not mid-life was predictive of dementia in older participants |
|  | Andrew & Rockwood, 2010^91^ | Magnitude of cognitive decline was linked with social impairment. Social vulnerability was associated with risk of cognitive decline |
|  | Andel et al, 2012^92^ | Increased dementia risk associated with lower social support & work-related stress |
|  | Sundstrom et al, 2014^93^ | Being widowed or childless was associated with dementia risk in older participants |
|  | Camozzato et al, 2015^94^ | Increased dementia risk associated with not having a confidante in older participants |
|  | Sundstrom et al, 2016^95^ | Increased risk of dementia was associated being widowed or divorced in young & middle-old participants |
|  | Khondoker et al, 2017^96^ | Dementia risk was associated with negative social interactions & lack of social support in older participants |
|  | Crooks et al, 2008^97^ | Reduced risk of dementia associated with larger social network & amount of social contact in older participants |
|  | Pedrosa et al, 2010^21^ | Talking about current events was impaired in MCI |
|  | Akbaraly et al, 2009^98^ | Social leisure activities were associated with reduced dementia risk |
|  | Chen et al, 2011^99^ | Older participants Subjects living alone were at greater risk of dementia than those living with family |
|  | Tripathi et al, 2012^100^ | Social engagement was protective against dementia risk |
|  | Wallin et al, 2013^101^ | Longitudinal risk of dementia was associated with baseline social contact |
|  | Fankhauser et al, 2015^102^ | MCI->AD conversion was reduced in those who visit friends regularly |
|  | Vemuri et al, 2016^33^ | In older HC & MCI, those with higher levels of education, increased midlife cognitive activity (including social activities) was associated with lower amyloid deposition in APOE4 homozygotes |
|  | Smit et al, 2016^28^ | In residents with dementia, regardless of cognitive status, activity involvement (including helping others & conversation groups) significantly related to care relationship score & social relations. There was negative correlation between activity involvement & positive self-image. |
|  | Marioni et al, 2015^103^ | Higher social engagement was associated with reduced dementia risk |
| *Motivation, signs of apathy or withdrawal* | Vemuri et al, 2016^33^ | In older HC & MCI, those with higher levels of education, increased midlife cognitive activity (including group activities, social activities) was associated with lower amyloid deposition in APOE4 homozygotes |
|  | Smit et al, 2016^28^ | In residents with dementia, regardless of cognitive status, activity involvement (including spiritual/religious activities, excursion or shopping, walking outside, cooking, conversation groups, sensory stimulation, beauty activities) positive affect, restless/tense behaviour & 'having something to do'. There was negative correlation between activity involvement & positive self-image |
|  | Tung et al, 2014^11^ | Life space correlated with apathy & depression in AD participants comparative to HC |

# References

1. Myung W, Lee C, Park JH, Woo SY, Kim S, Kim S, Chung JW, Kang HS, Lim SW, Choi J, Na DL, Kim SY, Lee JH, Han SH, Choi SH, Kim SY, Carroll BJ, Kim DK. Occupational Attainment as Risk Factor for Progression from Mild Cognitive Impairment to Alzheimer's Disease: A CREDOS Study. J Alzheimers Dis. 2017;55(1):283-292. doi: 10.3233/JAD-160257. PMID: 27662289.

2. Forstmeier S, Maercker A, Maier W, van den Bussche H, Riedel-Heller S, Kaduszkiewicz H, Pentzek M, Weyerer S, Bickel H, Tebarth F, Luppa M, Wollny A, Wiese B, Wagner M; AgeCoDe Study Group. Motivational reserve: motivation-related occupational abilities and risk of mild cognitive impairment and Alzheimer disease. Psychol Aging. 2012 Jun;27(2):353-63. doi: 10.1037/a0025117. Epub 2011 Aug 29. PMID: 21875213.

3. Boots EA, Schultz SA, Almeida RP, Oh JM, Koscik RL, Dowling MN, Gallagher CL, Carlsson CM, Rowley HA, Bendlin BB, Asthana S, Sager MA, Hermann BP, Johnson SC, Okonkwo OC. Occupational Complexity and Cognitive Reserve in a Middle-Aged Cohort at Risk for Alzheimer's Disease. Arch Clin Neuropsychol. 2015 Nov;30(7):634-42. doi: 10.1093/arclin/acv041. Epub 2015 Jul 8. PMID: 26156334; PMCID: PMC4605365.

4. Garibotto V, Borroni B, Kalbe E, Herholz K, Salmon E, Holtoff V, Sorbi S, Cappa SF, Padovani A, Fazio F, Perani D. Education and occupation as proxies for reserve in aMCI converters and AD: FDG-PET evidence. Neurology. 2008 Oct 21;71(17):1342-9. doi: 10.1212/01.wnl.0000327670.62378.c0. PMID: 18936426.

5. Tu S, Wong S, Hodges JR, Irish M, Piguet O, Hornberger M. Lost in spatial translation - A novel tool to objectively assess spatial disorientation in Alzheimer's disease and frontotemporal dementia. Cortex. 2015 Jun;67:83-94. doi: 10.1016/j.cortex.2015.03.016. Epub 2015 Apr 2. PMID: 25913063.

6. Yew B, Alladi S, Shailaja M, Hodges JR, Hornberger M. Lost and forgotten? Orientation versus memory in Alzheimer's disease and frontotemporal dementia. J Alzheimers Dis. 2013;33(2):473-81. doi: 10.3233/JAD-2012-120769. PMID: 22986775.7. Serino, S. *et al.* Out of body, out of space: Impaired reference frame processing in eating disorders. *Psychiatry Res.* (2015). doi:10.1016/j.psychres.2015.10.025

8. Lithfous S, Dufour A, Després O. Spatial navigation in normal aging and the prodromal stage of Alzheimer's disease: insights from imaging and behavioral studies. Ageing Res Rev. 2013 Jan;12(1):201-13. doi: 10.1016/j.arr.2012.04.007. Epub 2012 Jul 5. PMID: 22771718.

9. Mokrisova I, Laczo J, Andel R, Gazova I, Vyhnalek M, Nedelska Z, Levcik D, Cerman J, Vlcek K, Hort J. Real-space path integration is impaired in Alzheimer's disease and mild cognitive impairment. Behav Brain Res. 2016 Jul 1;307:150-8. doi: 10.1016/j.bbr.2016.03.052. Epub 2016 Mar 30. PMID: 27038766.

10. James, B. D., Wilson, R. S., Barnes, L. L., & Bennett, D. A. (2011). Late-life social activity and cognitive decline in old age. Journal of the International Neuropsychological Society : JINS, 17(6), 998–1005. https://doi.org/10.1017/S1355617711000531

11. Tung JY, Rose RV, Gammada E, Lam I, Roy EA, Black SE, Poupart P. Measuring life space in older adults with mild-to-moderate Alzheimer's disease using mobile phone GPS. Gerontology. 2014;60(2):154-62. doi: 10.1159/000355669. Epub 2013 Dec 12. PMID: 24356464.

12. Chan D, Gallaher LM, Moodley K, Minati L, Burgess N, Hartley T. The 4 Mountains Test: A Short Test of Spatial Memory with High Sensitivity for the Diagnosis of Pre-dementia Alzheimer's Disease. J Vis Exp. 2016 Oct 13;(116):54454. doi: 10.3791/54454. PMID: 27768046; PMCID: PMC5092189.

13. Moodley K, Minati L, Contarino V, Prioni S, Wood R, Cooper R, D'Incerti L, Tagliavini F, Chan D. Diagnostic differentiation of mild cognitive impairment due to Alzheimer's disease using a hippocampus-dependent test of spatial memory. Hippocampus. 2015 Aug;25(8):939-51. doi: 10.1002/hipo.22417. Epub 2015 Mar 26. PMID: 25605659.

14. Kalová E, Vlcek K, Jarolímová E, Bures J. Allothetic orientation and sequential ordering of places is impaired in early stages of Alzheimer's disease: corresponding results in real space tests and computer tests. Behav Brain Res. 2005 Apr 30;159(2):175-86. doi: 10.1016/j.bbr.2004.10.016. Epub 2004 Dec 7. PMID: 15817181.

15. Hort J, Andel R, Mokrisova I, Gazova I, Amlerova J, Valis M, Coulson EJ, Harrison J, Windisch M, Laczó J. Effect of donepezil in Alzheimer disease can be measured by a computerized human analog of the Morris water maze. Neurodegener Dis. 2014;13(2-3):192-6. doi: 10.1159/000355517. Epub 2013 Oct 30. PMID: 24192578.

16. Baumard J, Lesourd M, Jarry C, Merck C, Etcharry-Bouyx F, Chauviré V, Belliard S, Moreaud O, Croisile B, Osiurak F, Le Gall D. Tool use disorders in neurodegenerative diseases: Roles of semantic memory and technical reasoning. Cortex. 2016 Sep;82:119-132. doi: 10.1016/j.cortex.2016.06.007. Epub 2016 Jun 21. PMID: 27376932.

17. Schmitter-Edgecombe M, Parsey CM. Assessment of functional change and cognitive correlates in the progression from healthy cognitive aging to dementia. Neuropsychology. 2014 Nov;28(6):881-93. doi: 10.1037/neu0000109. Epub 2014 Jun 16. PMID: 24933485; PMCID: PMC4227927.

18. Lau KM, Parikh M, Harvey DJ, Huang CJ, Farias ST. Early Cognitively Based Functional Limitations Predict Loss of Independence in Instrumental Activities of Daily Living in Older Adults. J Int Neuropsychol Soc. 2015 Oct;21(9):688-98. doi: 10.1017/S1355617715000818. Epub 2015 Sep 22. PMID: 26391766; PMCID: PMC5540650.

19. Lawton MP, Brody EM. Assessment of older people: self-maintaining and instrumental activities of daily living. Gerontologist. 1969 Autumn;9(3):179-86. PMID: 5349366.

20. Farias ST, Lau K, Harvey D, Denny KG, Barba C, Mefford AN. Early Functional Limitations in Cognitively Normal Older Adults Predict Diagnostic Conversion to Mild Cognitive Impairment. J Am Geriatr Soc. 2017 Jun;65(6):1152-1158. doi: 10.1111/jgs.14835. Epub 2017 Mar 17. PMID: 28306147; PMCID: PMC5478464.

21. Pedrosa H, De Sa A, Guerreiro M, Maroco J, Simoes MR, Galasko D, de Mendonca A. Functional evaluation distinguishes MCI patients from healthy elderly people--the ADCS/MCI/ADL scale. J Nutr Health Aging. 2010 Oct;14(8):703-9. doi: 10.1007/s12603-010-0102-1. PMID: 20922349.

22. Barberger-Gateau P, Fabrigoule C, Helmer C, Rouch I, Dartigues JF. Functional impairment in instrumental activities of daily living: an early clinical sign of dementia? J Am Geriatr Soc. 1999 Apr;47(4):456-62. doi: 10.1111/j.1532-5415.1999.tb07239.x. PMID: 10203122.

23. Ashraf A, Taati B. Automated Video Analysis of Handwashing Behavior as a Potential Marker of Cognitive Health in Older Adults. IEEE J Biomed Health Inform. 2016 Mar;20(2):682-90. doi: 10.1109/JBHI.2015.2413358. Epub 2015 Mar 16. PMID: 25794404.

24. V. Buso, L. Hopper, J. Benois-Pineau, P. Plans and R. Mégret, "Recognition of Activities of Daily Living in natural “at home” scenario for assessment of Alzheimer's disease patients," 2015 IEEE International Conference on Multimedia & Expo Workshops (ICMEW), Turin, 2015, pp. 1-6, doi: 10.1109/ICMEW.2015.7169861.

25. Cortes F, Nourhashémi F, Guérin O, Cantet C, Gillette-Guyonnet S, Andrieu S, Ousset PJ, Vellas B; REAL-FR Group. Prognosis of Alzheimer's disease today: a two-year prospective study in 686 patients from the REAL-FR Study. Alzheimers Dement. 2008 Jan;4(1):22-9. doi: 10.1016/j.jalz.2007.10.018. PMID: 18631947.

26. Katz S, Downs TD, Cash HR, Grotz RC. Progress in development of the index of ADL. Gerontologist. 1970 Spring;10(1):20-30. doi: 10.1093/geront/10.1_part_1.20. PMID: 5420677.

27. Gillette-Guyonnet S, Andrieu S, Nourhashemi F, Gardette V, Coley N, Cantet C, Gauthier S, Ousset PJ, Vellas B; REAL.FR study group. Long-term progression of Alzheimer's disease in patients under antidementia drugs. Alzheimers Dement. 2011 Nov;7(6):579-92. doi: 10.1016/j.jalz.2011.02.009. PMID: 22055975.

28. Smit D, de Lange J, Willemse B, Twisk J, Pot AM. Activity involvement and quality of life of people at different stages of dementia in long term care facilities. Aging Ment Health. 2016;20(1):100-9. doi: 10.1080/13607863.2015.1049116. Epub 2015 Jun 2. PMID: 26032736.

29. McDermott KL, McFall GP, Andrews SJ, Anstey KJ, Dixon RA. Memory Resilience to Alzheimer's Genetic Risk: Sex Effects in Predictor Profiles. J Gerontol B Psychol Sci Soc Sci. 2017 Oct 1;72(6):937-946. doi: 10.1093/geronb/gbw161. PMID: 28025282; PMCID: PMC5927155.

30. Hedman, A., Lindqvist, E., & Nygård, L. (2016). How older adults with mild cognitive impairment relate to technology as part of present and future everyday life: a qualitative study. BMC geriatrics, 16, 73. https://doi.org/10.1186/s12877-016-0245-y

31. Law LL, Barnett F, Yau MK, Gray MA. Development and initial testing of functional task exercise on older adults with cognitive impairment at risk of Alzheimer's disease--FcTSim programme--a feasibility study. Occup Ther Int. 2013 Dec;20(4):185-97. doi: 10.1002/oti.1355. Epub 2013 Jun 13. PMID: 23761291.

32. Lancioni GE, Singh NN, O'Reilly MF, Sigafoos J, Renna C, Pinto K, De Vanna F, Caffò AO, Stasolla F. Persons with moderate Alzheimer's disease use simple technology aids to manage daily activities and leisure occupation. Res Dev Disabil. 2014 Sep;35(9):2117-28. doi: 10.1016/j.ridd.2014.05.002. Epub 2014 May 28. PMID: 24881006.

33. Vemuri P, Lesnick TG, Przybelski SA, Knopman DS, Machulda M, Lowe VJ, Mielke MM, Roberts RO, Gunter JL, Senjem ML, Geda YE, Rocca WA, Petersen RC, Jack CR Jr. Effect of intellectual enrichment on AD biomarker trajectories: Longitudinal imaging study. Neurology. 2016 Mar 22;86(12):1128-35. doi: 10.1212/WNL.0000000000002490. Epub 2016 Feb 24. PMID: 26911640; PMCID: PMC4820132.

34. Beaulieu-Bonneau S, Hudon C. Sleep disturbances in older adults with mild cognitive impairment. Int Psychogeriatr. 2009 Aug;21(4):654-66. doi: 10.1017/S1041610209009120. Epub 2009 May 11. PMID: 19426575.

35. Hatfield CF, Herbert J, van Someren EJ, Hodges JR, Hastings MH. Disrupted daily activity/rest cycles in relation to daily cortisol rhythms of home-dwelling patients with early Alzheimer's dementia. Brain. 2004 May;127(Pt 5):1061-74. doi: 10.1093/brain/awh129. Epub 2004 Mar 3. PMID: 14998915.

36. Gehrman P, Marler M, Martin JL, Shochat T, Corey-Bloom J, Ancoli-Israel S. The relationship between dementia severity and rest/activity circadian rhythms. Neuropsychiatr Dis Treat. 2005 Jun;1(2):155-63. doi: 10.2147/nedt.1.2.155.61043. PMID: 18568061; PMCID: PMC2413196.

37. Carvalho-Bos SS, Riemersma-van der Lek RF, Waterhouse J, Reilly T, Van Someren EJ. Strong association of the rest-activity rhythm with well-being in demented elderly women. Am J Geriatr Psychiatry. 2007 Feb;15(2):92-100. doi: 10.1097/01.JGP.0000236584.03432.dc. PMID: 17272729.

38. Carvalho-Bos SS, Riemersma-van der Lek RF, Waterhouse J, Reilly T, Van Someren EJ. Strong association of the rest-activity rhythm with well-being in demented elderly women. Am J Geriatr Psychiatry. 2007 Feb;15(2):92-100. doi: 10.1097/01.JGP.0000236584.03432.dc. PMID: 17272729.

39. Otsuka A, Mikami H, Katahira K, Nakamoto Y, Minamitani K, Imaoka M, Nishide M, Ogihara T. Absence of nocturnal fall in blood pressure in elderly persons with Alzheimer-type dementia. J Am Geriatr Soc. 1990 Sep;38(9):973-8. doi: 10.1111/j.1532-5415.1990.tb04418.x. PMID: 2212450.

40. Chen Q, Wang J, Tian J, Tang X, Yu C, Marshall RJ, Chen D, Cao W, Zhan S, Lv J, Lee L, Hu Y. Association between ambient temperature and blood pressure and blood pressure regulators: 1831 hypertensive patients followed up for three years. PLoS One. 2013 Dec 31;8(12):e84522. doi: 10.1371/journal.pone.0084522. PMID: 24391962; PMCID: PMC3877276.

41. Harper DG, Stopa EG, McKee AC, Satlin A, Fish D, Volicer L. Dementia severity and Lewy bodies affect circadian rhythms in Alzheimer disease. Neurobiol Aging. 2004 Jul;25(6):771-81. doi: 10.1016/j.neurobiolaging.2003.04.009. PMID: 15165702.

42. Volicer L, Harper DG, Manning BC, Goldstein R, Satlin A. Sundowning and circadian rhythms in Alzheimer's disease. Am J Psychiatry. 2001 May;158(5):704-11. doi: 10.1176/appi.ajp.158.5.704. PMID: 11329390.

43. Most EI, Aboudan S, Scheltens P, Van Someren EJ. Discrepancy between subjective and objective sleep disturbances in early- and moderate-stage Alzheimer disease. Am J Geriatr Psychiatry. 2012 Jun;20(6):460-7. doi: 10.1097/JGP.0b013e318252e3ff. PMID: 22531105.

44. Yamadera H, Ito T, Suzuki H, Asayama K, Ito R, Endo S. Effects of bright light on cognitive and sleep-wake (circadian) rhythm disturbances in Alzheimer-type dementia. Psychiatry Clin Neurosci. 2000 Jun;54(3):352-3. doi: 10.1046/j.1440-1819.2000.00711.x. PMID: 11186110.

45. Satlin A, Volicer L, Ross V, Herz L, Campbell S. Bright light treatment of behavioral and sleep disturbances in patients with Alzheimer's disease. Am J Psychiatry. 1992 Aug;149(8):1028-32. doi: 10.1176/ajp.149.8.1028. PMID: 1353313.

46. Prinz, P. N., Vitaliano, P. P., Vitiello, M. V., Bokan, J., Raskind, M., Peskind, E., & Gerber, C. (1982). Sleep, EEG and mental function changes in senile dementia of the Alzheimer's type. Neurobiology of Aging, 3(4), 361–370.

47. Jutten RJ, Peeters CFW, Leijdesdorff SMJ, Visser PJ, Maier AB, Terwee CB, Scheltens P, Sikkes SAM. Detecting functional decline from normal aging to dementia: Development and validation of a short version of the Amsterdam IADL Questionnaire. Alzheimers Dement (Amst). 2017 Mar 31;8:26-35. doi: 10.1016/j.dadm.2017.03.002. PMID: 28462387; PMCID: PMC5403784.

48. Stringer G, Couth S, Brown LJE, Montaldi D, Gledson A, Mellor J, Sutcliffe A, Sawyer P, Keane J, Bull C, Zeng X, Rayson P, Leroi I. Can you detect early dementia from an email? A proof of principle study of daily computer use to detect cognitive and functional decline. Int J Geriatr Psychiatry. 2018 Jul;33(7):867-874. doi: 10.1002/gps.4863. Epub 2018 Feb 9. PMID: 29424087; PMCID: PMC6033108.

49. Hedman A, Kottorp A, Almkvist O, Nygård L. Challenge levels of everyday technologies as perceived over five years by older adults with mild cognitive impairment. Int Psychogeriatr. 2018 Oct;30(10):1447-1454. doi: 10.1017/S1041610218000285. Epub 2018 Apr 4. PMID: 29615145; PMCID: PMC6317289.

50. Malinowsky C, Kottorp A, Wallin A, Nordlund A, Björklund E, Melin I, Pernevik A, Rosenberg L, Nygård L. Differences in the use of everyday technology among persons with MCI, SCI and older adults without known cognitive impairment. Int Psychogeriatr. 2017 Jul;29(7):1193-1200. doi: 10.1017/S1041610217000643. Epub 2017 Apr 17. PMID: 28412981.

51. Couth S, Stringer G, Leroi I, et al. Which computer-use behaviours are most indicative of cognitive decline? Insights from an expert reference group. Health Informatics Journal. 2019;25(3):1053-1064. doi:10.1177/1460458217739342

52. Seelye A, Hagler S, Mattek N, Howieson DB, Wild K, Dodge HH, Kaye JA. Computer mouse movement patterns: A potential marker of mild cognitive impairment. Alzheimers Dement (Amst). 2015 Dec 1;1(4):472-480. doi: 10.1016/j.dadm.2015.09.006. Epub 2015 Oct 19. PMID: 26878035; PMCID: PMC4748737.

53. Austin, J., Hollingshead, K., & Kaye, J. (2017). Internet Searches and Their Relationship to Cognitive Function in Older Adults: Cross-Sectional Analysis. Journal of medical Internet research, 19(9), e307. https://doi.org/10.2196/jmir.7671

54. Geda, Y. E., Topazian, H. M., Roberts, L. A., Roberts, R. O., Knopman, D. S., Pankratz, V. S., Christianson, T. J., Boeve, B. F., Tangalos, E. G., Ivnik, R. J., & Petersen, R. C. (2011). Engaging in cognitive activities, aging, and mild cognitive impairment: a population-based study. The Journal of neuropsychiatry and clinical neurosciences, 23(2), 149–154. https://doi.org/10.1176/jnp.23.2.jnp149

55. Anstey KJ, Cherbuin N, Eramudugolla R, Sargent-Cox K, Easteal S, Kumar R, Sachdev P. Characterizing mild cognitive disorders in the young-old over 8 years: prevalence, estimated incidence, stability of diagnosis, and impact on IADLs. Alzheimers Dement. 2013 Nov;9(6):640-8. doi: 10.1016/j.jalz.2012.11.013. Epub 2013 Mar 7. PMID: 23474041.

56. Ellendt S, Voβ B, Kohn N, Wagels L, Goerlich KS, Drexler E, Schneider F, Habel U. Predicting Stability of Mild Cognitive Impairment (MCI): Findings of a Community Based Sample. Curr Alzheimer Res. 2017;14(6):608-619. doi: 10.2174/1567205014666161213120807. PMID: 27978792.

57. Mesulam, M., Wicklund, A., Johnson, N., Rogalski, E., Léger, G. C., Rademaker, A., Weintraub, S., & Bigio, E. H. (2008). Alzheimer and frontotemporal pathology in subsets of primary progressive aphasia. Annals of neurology, 63(6), 709–719. https://doi.org/10.1002/ana.21388

58. Nutter-Upham, K. E., Saykin, A. J., Rabin, L. A., Roth, R. M., Wishart, H. A., Pare, N., & Flashman, L. A. (2008). Verbal fluency performance in amnestic MCI and older adults with cognitive complaints. Archives of clinical neuropsychology : the official journal of the National Academy of Neuropsychologists, 23(3), 229–241. https://doi.org/10.1016/j.acn.2008.01.005

59. Bracco L, Gallato R, Grigoletto F, Lippi A, Lepore V, Bino G, Lazzaro MP, Carella F, Piccolo T, Pozzilli C, et al. Factors affecting course and survival in Alzheimer's disease. A 9-year longitudinal study. Arch Neurol. 1994 Dec;51(12):1213-9. doi: 10.1001/archneur.1994.00540240057016. PMID: 7986176.

60. Bilgel, M., An, Y., Lang, A., Prince, J., Ferrucci, L., Jedynak, B., & Resnick, S. M. (2014). Trajectories of Alzheimer disease-related cognitive measures in a longitudinal sample. Alzheimer's & dementia : the journal of the Alzheimer's Association, 10(6), 735–742.e4. https://doi.org/10.1016/j.jalz.2014.04.520

61. Yotter RA, Doshi J, Clark V, Sojkova J, Zhou Y, Wong DF, Ferrucci L, Resnick SM, Davatzikos C. Memory decline shows stronger associations with estimated spatial patterns of amyloid deposition progression than total amyloid burden. Neurobiol Aging. 2013 Dec;34(12):2835-42. doi: 10.1016/j.neurobiolaging.2013.05.030. Epub 2013 Jul 13. PMID: 23859610; PMCID: PMC3893024.

62. Tang-Wai, D. F. & Graham, N. L. Assessment of language function in dementia. *Geriatrics and Aging* (2008) 11(2):103-110.

63. Manenti R, Repetto C, Bentrovato S, Marcone A, Bates E, Cappa SF. The effects of ageing and Alzheimer's disease on semantic and gender priming. Brain. 2004 Oct;127(Pt 10):2299-306. doi: 10.1093/brain/awh264. Epub 2004 Aug 11. PMID: 15306548.

64. Nadkarni NK, McIlroy WE, Mawji E, Black SE. Gait and subcortical hyperintensities in mild Alzheimer's disease and aging. Dement Geriatr Cogn Disord. 2009;28(4):295-301. doi: 10.1159/000245158. Epub 2009 Oct 10. PMID: 19828950.

65. Ansai JH, Andrade LP, Masse FAA, Gonçalves J, Takahashi ACM, Vale FAC, Rebelatto JR. Risk Factors for Falls in Older Adults With Mild Cognitive Impairment and Mild Alzheimer Disease. J Geriatr Phys Ther. 2019 Jul/Sep;42(3):E116-E121. doi: 10.1519/JPT.0000000000000135. PMID: 28786910.

66. Mazoteras Muñoz V, Abellan van Kan G, Cantet C, Cortes F, Ousset PJ, Rolland Y, Vellas B. Gait and balance impairments in Alzheimer disease patients. Alzheimer Dis Assoc Disord. 2010 Jan-Mar;24(1):79-84. doi: 10.1097/WAD.0b013e3181c78a20. PMID: 20220324.

67. Montero-Odasso M, Casas A, Hansen KT, Bilski P, Gutmanis I, Wells JL, Borrie MJ. Quantitative gait analysis under dual-task in older people with mild cognitive impairment: a reliability study. J Neuroeng Rehabil. 2009 Sep 21;6:35. doi: 10.1186/1743-0003-6-35. PMID: 19772593; PMCID: PMC2754991.

68. Maquet D, Lekeu F, Warzee E, Gillain S, Wojtasik V, Salmon E, Petermans J, Croisier JL. Gait analysis in elderly adult patients with mild cognitive impairment and patients with mild Alzheimer's disease: simple versus dual task: a preliminary report. Clin Physiol Funct Imaging. 2010 Jan;30(1):51-6. doi: 10.1111/j.1475-097X.2009.00903.x. Epub 2009 Oct 2. PMID: 19799614.

69. Beauchet O, Allali G, Thiery S, Gautier J, Fantino B, Annweiler C. Association between high variability of gait speed and mild cognitive impairment: a cross-sectional pilot study. J Am Geriatr Soc. 2011 Oct;59(10):1973-4. doi: 10.1111/j.1532-5415.2011.03610_9.x. Erratum in: J Am Geriatr Soc. 2012 Oct;60(10):2001. PMID: 22091517.

70. Montero-Odasso M, Muir SW, Speechley M. Dual-task complexity affects gait in people with mild cognitive impairment: the interplay between gait variability, dual tasking, and risk of falls. Arch Phys Med Rehabil. 2012 Feb;93(2):293-9. doi: 10.1016/j.apmr.2011.08.026. PMID: 22289240.

71. Muir SW, Speechley M, Wells J, Borrie M, Gopaul K, Montero-Odasso M. Gait assessment in mild cognitive impairment and Alzheimer's disease: the effect of dual-task challenges across the cognitive spectrum. Gait Posture. 2012 Jan;35(1):96-100. doi: 10.1016/j.gaitpost.2011.08.014. Epub 2011 Sep 22. PMID: 21940172.

72. Sheridan PL, Solomont J, Kowall N, Hausdorff JM. Influence of executive function on locomotor function: divided attention increases gait variability in Alzheimer's disease. J Am Geriatr Soc. 2003 Nov;51(11):1633-7. doi: 10.1046/j.1532-5415.2003.51516.x. PMID: 14687395.

73. Anderson SW, Aksan N, Dawson JD, Uc EY, Johnson AM, Rizzo M. Neuropsychological assessment of driving safety risk in older adults with and without neurologic disease. J Clin Exp Neuropsychol. 2012;34(9):895-905. doi: 10.1080/13803395.2011.630654. Epub 2012 Sep 3. PMID: 22943767; PMCID: PMC3910382.

74. Fernandez-Romero, R. & Cox, D. J. IMPAIRED DRIVING CAPACITY IN EARLY STAGE ALZHEIMER’S IS ASSOCIATED WITH DECREASED CORTICAL RESPONSIVENESS TO SIMULATED SELF-MOVEMENT. *Alzheimer’s Dement.* (2016) 12:882. doi:10.1016/j.jalz.2016.06.1824

75. Wadley VG, Okonkwo O, Crowe M, Vance DE, Elgin JM, Ball KK, Owsley C. Mild cognitive impairment and everyday function: an investigation of driving performance. J Geriatr Psychiatry Neurol. 2009 Jun;22(2):87-94. doi: 10.1177/0891988708328215. Epub 2009 Feb 4. PMID: 19196629; PMCID: PMC2832580.

76. Paire-Ficout L, Lafont S, Conte F, Coquillat A, Fabrigoule C, Ankri J, Blanc F, Gabel C, Novella JL, Morrone I, Mahmoudi R. Naturalistic Driving Study Investigating Self-Regulation Behavior in Early Alzheimer's Disease: A Pilot Study. J Alzheimers Dis. 2018;63(4):1499-1508. doi: 10.3233/JAD-171031. PMID: 29782312.

77. Roe CM, Barco PP, Head DM, Ghoshal N, Selsor N, Babulal GM, Fierberg R, Vernon EK, Shulman N, Johnson A, Fague S, Xiong C, Grant EA, Campbell A, Ott BR, Holtzman DM, Benzinger TL, Fagan AM, Carr DB, Morris JC. Amyloid Imaging, Cerebrospinal Fluid Biomarkers Predict Driving Performance Among Cognitively Normal Individuals. Alzheimer Dis Assoc Disord. 2017 Jan-Mar;31(1):69-72. doi: 10.1097/WAD.0000000000000154. PMID: 27128959; PMCID: PMC5085874.

78. Ott BR, Heindel WC, Papandonatos GD, Festa EK, Davis JD, Daiello LA, Morris JC. A longitudinal study of drivers with Alzheimer disease. Neurology. 2008 Apr 1;70(14):1171-8. doi: 10.1212/01.wnl.0000294469.27156.30. Epub 2008 Jan 23. PMID: 18216302; PMCID: PMC3664938.

79. Hird MA, Vesely KA, Fischer CE, Graham SJ, Naglie G, Schweizer TA. Investigating Simulated Driving Errors in Amnestic Single- and Multiple-Domain Mild Cognitive Impairment. J Alzheimers Dis. 2017;56(2):447-452. doi: 10.3233/JAD-160995. PMID: 27983557.

80. Ott BR, Heindel WC, Whelihan WM, Caron MD, Piatt AL, Noto RB. A single-photon emission computed tomography imaging study of driving impairment in patients with Alzheimer's disease. Dement Geriatr Cogn Disord. 2000 May-Jun;11(3):153-60. doi: 10.1159/000017229. PMID: 10765046; PMCID: PMC3292192.

81. Ott BR, Jones RN, Noto RB, Yoo DC, Snyder PJ, Bernier JN, Carr DB, Roe CM. Brain amyloid in preclinical Alzheimer's disease is associated with increased driving risk. Alzheimers Dement (Amst). 2016 Nov 29;6:136-142. doi: 10.1016/j.dadm.2016.10.008. PMID: 28239638; PMCID: PMC5318288.

82. Piersma D, Fuermaier AB, de Waard D, Davidse RJ, de Groot J, Doumen MJ, Bredewoud RA, Claesen R, Lemstra AW, Vermeeren A, Ponds R, Verhey F, Brouwer WH, Tucha O. Prediction of Fitness to Drive in Patients with Alzheimer's Dementia. PLoS One. 2016 Feb 24;11(2):e0149566. doi: 10.1371/journal.pone.0149566. PMID: 26910535; PMCID: PMC4766198.

83. Bora E, Yener GG. Meta-Analysis of Social Cognition in Mild Cognitive Impairment. J Geriatr Psychiatry Neurol. 2017 Jul;30(4):206-213. doi: 10.1177/0891988717710337. PMID: 28639876.

84. Lavenu I, Pasquier F. Perception of emotion on faces in frontotemporal dementia and Alzheimer's disease: a longitudinal study. Dement Geriatr Cogn Disord. 2005;19(1):37-41. doi: 10.1159/000080969. Epub 2004 Sep 21. PMID: 15383744.

85. Phillips LH, Scott C, Henry JD, Mowat D, Bell JS. Emotion perception in Alzheimer's disease and mood disorder in old age. Psychol Aging. 2010 Mar;25(1):38-47. doi: 10.1037/a0017369. PMID: 20230126.

86. Albert MS, Cohen C, Koff E. Perception of affect in patients with dementia of the Alzheimer type. Arch Neurol. 1991 Aug;48(8):791-5. doi: 10.1001/archneur.1991.00530200027013. PMID: 1898252.

87. Bediou B, Ryff I, Mercier B, Milliery M, Hénaff MA, D'Amato T, Bonnefoy M, Vighetto A, Krolak-Salmon P. Impaired social cognition in mild Alzheimer disease. J Geriatr Psychiatry Neurol. 2009 Jun;22(2):130-40. doi: 10.1177/0891988709332939. Epub 2009 Mar 25. PMID: 19321881.

88. Bertoux M, de Souza LC, Sarazin M, Funkiewiez A, Dubois B, Hornberger M. How Preserved is Emotion Recognition in Alzheimer Disease Compared With Behavioral Variant Frontotemporal Dementia? Alzheimer Dis Assoc Disord. 2015 Apr-Jun;29(2):154-7. doi: 10.1097/WAD.0000000000000023. PMID: 24614268.

89. Zhang, X., Li, C. & Zhang, M. [Psychosocial risk factors of Alzheimer’s disease]. Zhonghua Yi Xue Za Zhi (1999) May;79(5):335-8. PMID: 11715471.

90. He, Y. L., Zhang, X. K. & Zhang, M. Y. Psychosocial risk factors for Alzheimer’s disease. *Hong Kong J. Psychiatry* **10**, 2–7 (2000)..

91. Andrew MK, Rockwood K. Social vulnerability predicts cognitive decline in a prospective cohort of older Canadians. Alzheimers Dement. 2010 Jul;6(4):319-325.e1. doi: 10.1016/j.jalz.2009.11.001. PMID: 20630414.

92. Andel R, Crowe M, Hahn EA, Mortimer JA, Pedersen NL, Fratiglioni L, Johansson B, Gatz M. Work-related stress may increase the risk of vascular dementia. J Am Geriatr Soc. 2012 Jan;60(1):60-7. doi: 10.1111/j.1532-5415.2011.03777.x. Epub 2011 Dec 16. PMID: 22175444; PMCID: PMC3258308.

93. Sundström A, Westerlund O, Mousavi-Nasab H, Adolfsson R, Nilsson LG. The relationship between marital and parental status and the risk of dementia. Int Psychogeriatr. 2014 May;26(5):749-57. doi: 10.1017/S1041610213002652. Epub 2014 Jan 22. PMID: 24451183.

94. Camozzato A, Godinho C, Varela J, Kohler C, Rinaldi J, Chaves ML. The complex role of having confidant on the development of Alzheimer's disease in a community-based cohort of older people in Brazil. Neuroepidemiology. 2015;44(2):78-82. doi: 10.1159/000371521. Epub 2015 Mar 5. PMID: 25765048.

95. Sundström A, Westerlund O, Kotyrlo E. Marital status and risk of dementia: a nationwide population-based prospective study from Sweden. BMJ Open. 2016 Jan 4;6(1):e008565. doi: 10.1136/bmjopen-2015-008565. PMID: 26729377; PMCID: PMC4716184.

96. Khondoker M, Rafnsson SB, Morris S, Orrell M, Steptoe A. Positive and Negative Experiences of Social Support and Risk of Dementia in Later Life: An Investigation Using the English Longitudinal Study of Ageing. J Alzheimers Dis. 2017;58(1):99-108. doi: 10.3233/JAD-161160. PMID: 28387667; PMCID: PMC5438469.

97. Crooks VC, Lubben J, Petitti DB, Little D, Chiu V. Social network, cognitive function, and dementia incidence among elderly women. Am J Public Health. 2008 Jul;98(7):1221-7. doi: 10.2105/AJPH.2007.115923. Epub 2008 May 29. PMID: 18511731; PMCID: PMC2424087.

98. Akbaraly TN, Portet F, Fustinoni S, Dartigues JF, Artero S, Rouaud O, Touchon J, Ritchie K, Berr C. Leisure activities and the risk of dementia in the elderly: results from the Three-City Study. Neurology. 2009 Sep 15;73(11):854-61. doi: 10.1212/WNL.0b013e3181b7849b. PMID: 19752452.

99. Chen R, Hu Z, Wei L, Ma Y, Liu Z, Copeland JR. Incident dementia in a defined older Chinese population. PLoS One. 2011;6(9):e24817. doi: 10.1371/journal.pone.0024817. Epub 2011 Sep 23. PMID: 21966372; PMCID: PMC3179466.

100. Tripathi M, Vibha D, Gupta P, Bhatia R, Srivastava MV, Vivekanandhan S, Bhushan Singh M, Prasad K, Dergalust S, Mendez MF. Risk factors of dementia in North India: a case-control study. Aging Ment Health. 2012;16(2):228-35. doi: 10.1080/13607863.2011.583632. Epub 2011 Jun 30. PMID: 21714688. doi:10.1080/13607863.2011.583632

101. Wallin K, Boström G, Kivipelto M, Gustafson Y. Risk factors for incident dementia in the very old. Int Psychogeriatr. 2013 Jul;25(7):1135-43. doi: 10.1017/S1041610213000409. Epub 2013 Apr 11. PMID: 23574921.

102. Fankhauser S, Forstmeier S, Maercker A, Luppa M, Luck T, Riedel-Heller SG. Risk of dementia in older adults with low versus high occupation-based motivational processes: differential impact of frequency and proximity of social network. J Geriatr Psychiatry Neurol. 2015 Jun;28(2):126-35. doi: 10.1177/0891988714554706. Epub 2014 Nov 26. PMID: 25431449.

103. Marioni RE, Proust-Lima C, Amieva H, Brayne C, Matthews FE, Dartigues JF, Jacqmin-Gadda H. Social activity, cognitive decline and dementia risk: a 20-year prospective cohort study. BMC Public Health. 2015 Oct 24;15:1089. doi: 10.1186/s12889-015-2426-6. PMID: 26499254; PMCID: PMC4619410.
