# Supplementary material for: Selecting Remote Measurement Technologies to Optimize Assessment of Function in Early Alzheimer's Disease: A Case Study
Source: Front Psychiatry. 2020 Nov 5;11:582207. doi: 10.3389/fpsyt.2020.582207 (PMC7674649; doi:10.3389/fpsyt.2020.582207)
Supplement: Supplementary file 2 [file Table_2.DOCX]

Supplementary Material 2

**Supplementary table 2.** Results from the RADAR-AD Patient Advisory Board’s workshop on functional domain relevance to people with dementia and carers

| Fairly and very important functional domains combined | | | |
| --- | --- | --- | --- |
| Carers/supporters | | **People with dementia** | |
| Score | **Functional domain** | **Score** | **Functional domain** |
| 10 | Keeping track of things | 10 | Learning new skills |
| 10 | Reading and writing | 10 | Using computer/phone |
| 10 | Managing finances | 10 | Sleeping |
| 10 | Navigating | 9 | Reading and writing |
| 10 | Planning & completing tasks | 9 | Keeping track of things |
| 9 | Organising self-care | 7 | Managing finances |
| 9 | At work | 7 | Navigating |
| 8 | Using computer/phone | 8 | Organising self-care |
| 8 | Misplacing objects | 7 | Household management |
| 7 | Driving | 7 | Planning and completing tasks |
| 7 | Sleeping | 6 | Understanding the plot |
| 8 | Household management | 8 | Finding the right words |
| 7 | Finding the right words | 5 | At work |
| 5 | Walking | 5 | Driving |
| 5 | Remembering recent events | 8 | Walking |
| 5 | Understanding the plot | 8 | Misplacing objects |
| 4 | Learning new skills | 8 | Remembering recent events |
